# Maternal Exposure to High-Fat Diet Induces Long-Term Derepressive Chromatin Marks in the Heart

**DOI:** 10.3390/nu12010181

**Published:** 2020-01-09

**Authors:** Guillaume Blin, Marjorie Liand, Claire Mauduit, Hassib Chehade, Mohamed Benahmed, Umberto Simeoni, Benazir Siddeek

**Affiliations:** 1 Woman-Mother-Child Department, Division of Pediatrics, DOHaD Laboratory, Centre Hospitalier Universitaire Vaudois and University of Lausanne, Rue du Bugnon 27, 1011 Lausanne, Switzerland; Guillaume.Blin@chuv.ch (G.B.); marjorie.liand@chuv.ch (M.L.); hassib.chehade@chuv.ch (H.C.); umberto.simeoni@chuv.ch (U.S.); 2 INSERM U1065, Centre Méditerranéen de Médecine Moléculaire (C3M), Team 5, 06204 Nice, France; claire.mauduit@univ-lyon1.fr (C.M.); Mohamed.benahmed@unice.fr (M.B.)

**Keywords:** heart, high-fat diet, development, polycomb repressive complex, DNA methylation

## Abstract

Heart diseases are a leading cause of death. While the link between early exposure to nutritional excess and heart disease risk is clear, the molecular mechanisms involved are poorly understood. In the developmental programming field, increasing evidence is pointing out the critical role of epigenetic mechanisms. Among them, polycomb repressive complex 2 (PRC2) and DNA methylation play a critical role in heart development and pathogenesis. In this context, we aimed at evaluating the role of these epigenetic marks in the long-term cardiac alterations induced by early dietary challenge. Using a model of rats exposed to maternal high-fat diet during gestation and lactation, we evaluated cardiac alterations at adulthood. Expression levels of PRC2 components, its histone marks di- and trimethylated histone H3 (H3K27me2/3), associated histone mark (ubiquitinated histone H2A, H2AK119ub1) and target genes were measured by Western blot. Global DNA methylation level and DNA methyl transferase 3B (DNMT3B) protein levels were measured. Maternal high-fat diet decreased H3K27me3, H2Ak119ub1 and DNA methylation levels, down-regulated the enhancer of zeste homolog 2 (EZH2), and DNMT3B expression. The levels of the target genes, isl lim homeobox 1 (*Isl1)*, six homeobox 1 (*Six1*) and mads box transcription enhancer factor 2, polypeptide C (*Mef2c)*, involved in cardiac pathogenesis were up regulated. Overall, our data suggest that the programming of cardiac alterations by maternal exposure to high-fat diet involves the derepression of pro-fibrotic and pro-hypertrophic genes through the induction of EZH2 and DNMT3B deficiency.

## 1. Introduction

A worldwide upward trend in the burden of non-communicable diseases (NCDs) with developmental origin such as obesity, diabetes, hypertension, cancers, infertility, immune, mental, and cardiovascular diseases is currently observed [1]. Epidemiological and experimental studies clearly indicate that environmental exposures such as lifestyle and diet, smoking, diabetes, obesity and exposure to toxicants increase the susceptibility to develop non-communicable, chronic diseases. Cardiovascular diseases (CVDs), which represent the main cause of death worldwide, are critically influenced by diet [2]. For instance, while increased risk for CVDs has been described with dietary patterns associated with urbanization, economic development and globalization, a well-tested healthy dietary pattern provided by a traditional Mediterranean diet has proven remarkable beneficial effects on CVDs [3]. Thus, dietary risk appears as a priority target for CVD prevention and treatment [3]. Gravely, the prevalence of obesity including maternal obesity has recently been increasing at an alarming rate. Obesity is often related to poor nutritional balance, with the consumption of food high in fat or sugar. Considering the limited cardiomyocytes turnover, the heart could be highly sensitive to early events like an exposure to over nutrition [4]. Indeed, early nutritional excess represents a risk factor for impaired cardiac function in adulthood by inducing an increased heart rate and left ventricular wall thickness, hypertrophy [5], fibrosis [6,7], and metabolic and hemodynamic heart impairment [8] due to early changes in cardiac gene expression [9]. While the link between maternal over-nutrition and heart disease risk is clear, the molecular basis of such programming of heart diseases is poorly understood. In the understanding of NCDs early origins, increasing evidence is pointing out the critical role of epigenetic mechanisms that are at stake during the perinatal period of development at the time when the embryo, the fetus and the infant undergo profound changes. Interestingly, such mechanisms were described as a bridge between nutrition and health [10]. Indeed, nutrients and bioactive food components can influence epigenetic phenomena either by directly inhibiting enzymes that catalyze DNA methylation or histone modifications, or by altering the availability of substrates necessary for those enzymatic reactions. In this regard, nutritional epigenetics is viewed as an attractive tool to prevent developmental diseases. The term epigenetic refers to the heritable changes in gene expression regulation without modification in the DNA sequence. The major epigenetic features include DNA methylation, post-translational histone modifications and RNA-based mechanisms which are tightly interrelated. During the heart development, these epigenetic mechanisms regulate the different steps involving coordinated cellular proliferation, migration, differentiation and programmed cell death, structural remodeling including looping and septation [11]. As a first step in the understanding of epigenetic programming of adult cardiac diseases by early exposure to nutritional excess, we previously highlighted the potential role of microRNAs in cardiac hypertrophy and fibrosis induced by maternal high-fat diet [12]. The decrease in DiGeorge syndrome critical region 8 (*Dgcr8*) expression, involved in microRNA biogenesis, seemed to be a critical point in the alterations of the microRNA profile and the transforming growth factor beta (TGFβ)-mediated remodeling induced by maternal exposure to high-fat diet [12]. However, DGCR8 deficiency did not explain the decreased levels of a few microRNAs which indicates that additional mechanisms might be involved in microRNAs regulation and in cardiac pathogenesis driven by maternal exposure to high-fat diet [12]. The accumulation of alterations in multiple epigenetic processes plays an important role in pathogenesis [13] with a greater impact on disease risk than genetic alterations in some tissues [14]. In the context of carcinogenic progression or during ageing, feedback loops can lead to ever-worsening epigenetic and/or genetic alterations [15,16]. Thus, the characterization of cumulative alterations in multiple epigenetic processes, including histone modifications and DNA methylation, appears as an important point to address to fully understand the pathogenic processes induced by early exposure to nutritional excess. Importantly, histone modifications have pervasive roles in the heart development. Regulation via histone methylation stabilizes transcriptional programs in embryonic progenitors and their differentiated descendants and is likely critical for establishing and maintaining gene expression and stress responses throughout life. During development, in addition to its role in transcriptional regulation, histone methylation has been proposed to exert its effects through the induction of mechanical changes [17]. Indeed, in bone marrow-derived mesenchymal stem cells, trimethylation of the lysine 27, on histone H3 (H3K27me3) has been suggested to induce chromatin condensation forming a nuclear skeleton that facilitates cell migration under chemokine (Osteopontin) stimulation [17]. The di- and especially trimethylation of the lysine 27, on histone H3 (H3K27me2/3) are well-described marks associated with the silencing of genes involved in main cellular processes [18]. The methylation is catalyzed by histone methyl transferases on amino acid residues of the histones. The reaction is realized by polycomb repressive complex 2 (PRC2) with core components: embryonic ectoderm development (EED), suppressor of zeste 12 (SUZ12) [19] and either enhancer zeste of homolog 1 or 2 (EZH1/2) [18,20]. The H3K27me3, then, provides a platform to recruit polycomb repressive complex 1 (PRC1) and DNA methyl transferases (DNMTs) which aid in polycomb protein-mediated repression either by chromatin compaction or by interfering with the transcription machinery. During heart development, PRC2 functions in the transition from cardiac progenitor to differentiated cardiomyocyte, and the critical role of EZH2 has been shown particularly in the stabilization of cardiac gene expression and pro-hypertrophic and pro-fibrotic genes [21]. In this context, the analysis of EZH2 and the associated chromatin repressive marks represent a potential candidate in the understanding of the long-term “developmental programming” effects induced by early exposure to nutritional excess on cardiac function. 

## 2. Materials and Methods 

### 2.1. Materials

Protease inhibitor cocktail, Tween 20, GeneElute Mammalian genomic DNA miniprep kit and other reagents were bought from Sigma Aldrich. Antibodies raised against Glyceraldehyde 3-phosphate dehydrogenase (GAPDH), β-Actin, anti-rabbit and anti-mouse immunoglobulin G horseradish peroxidase-linked antibody; Bicinchoninic acid Protein Assay Kit; Radioimmunoprecipitation assay buffer; phosphatase inhibitor cocktail; Chemiluminescent Western pico plus Substrate were bought from Thermofisher Scientific. PVDF membranes were bought from Amersham. Antibody raised against histone H3 dimethyl lysine 27 (H3K27me2) was bought from Cell Signaling Technology. Antibodies raised against isl lim homeobox 1 (ISL1), six homeobox 1 (SIX1) and mads box transcription enhancer factor 2, polypeptide C (MEF2C) were obtained from Santa Cruz Biotechnology. Enhancer of zeste homolog 2 (EZH2), histone H3 trimethyl lysine 27 (H3K27me3) and histone H3 antibodies were purchased from GeneTex. Suppressor of zeste 12 (SUZ12) antibody was bought from WuXi Biosciences. Anti-DNA methyl transferase 3B (DNMT3B) was bought from Abgent. MethylFlash Global DNA Methylation ELISA Easy kit was bought from Epigenetek.

### 2.2. In Vivo Experimental Studies

The animal experiment was performed in accordance with EU legislation (Directive 2010/63/EU) and approved by a local animal care and use committee (Comité Institutionnel d’Éthique Pour l’Animal de Laboratoire -CIEPAL-Azur- Agreement number: NCE-2013-109; Protocol No. 2015-62). Sprague Dawley rats (Janvier, Le Genest Saint Isle, France) were received at gestational day (GD) 7 and individually housed. They were either given a chow diet (R03, Safe, Augy, France, *n* = 5) or a high-fat diet (SNIFF 60%, SSNIFF, Soest, Germany, *n* = 5). Diet composition is described in supplementary information file. Litters were culled to 5 pups per dam at birth. At postnatal day (PND) 21, all male rats were fed with chow diet (R03). At PND 77, 18 male rats per group of diet were euthanized with CO_2_. The set of animals used in this study is the same as the one previously described [12].

### 2.3. Heart Sampling

Frozen tissues were grounded into powder for further molecular analyzes.

### 2.4. Protein Extraction 

In total, ~20 mg of frozen heart tissue was incubated with RIPA buffer (containing 1% proteases and phosphatases inhibitor cocktail). Protein concentration was measured. For Western blot analyses, 10 to 30 μg of protein was used.

### 2.5. Western Blotting Analysis

Proteins were loaded in SDS-PAGE gels and transferred to polyvinylidene difluoride membranes. The membranes were incubated with phosphate-buffered saline (pH 7.4) containing 0.05% Tween 20 and 1% BSA. Then, membranes were incubated with primary antibodies and horseradish peroxidase-conjugated secondary anti-rabbit or -mouse antibodies. SuperSignal West Pico PLUS Chemiluminescent Substrate was used for protein detection. A luminescent image analyzer camera G: Box (Syngene, Cambridge, UK) was used for luminescent signal scanning. The signals were quantified with Gene Tools software (Syngene, Cambridge, UK).

### 2.6. DNA Methylation 

Total DNA was isolated from frozen heart powder using the GeneElute Mammalian genomic DNA miniprep kit, according to the manufacturer’s protocol. The quantity of total DNA was evaluated with a spectrophotometer (Nanodrop). In total, 100 ng of isolated DNA was used for methylation analyses. The 5-Methyl Cytosine (5-mC) levels were measured using the MethylFlash Global DNA Methylation ELISA Easy kit, according to the manufacturer’s protocol.

### 2.7. Data Analysis

GraphPad Prism software version 6.05 (GraphPad Software, Inc.) was used for data analyses. The values were expressed as the mean ± SEM to account for variation between animals within a dataset. To determine whether there were differences between the two groups of diet, Student’s *t* test was performed. *p* < 0.05 was considered significant. 

## 3. Results

### 3.1. Exposure to Maternal High-Fat Diet Induces Long-Term Alterations in PRC2

**Figure 1:**
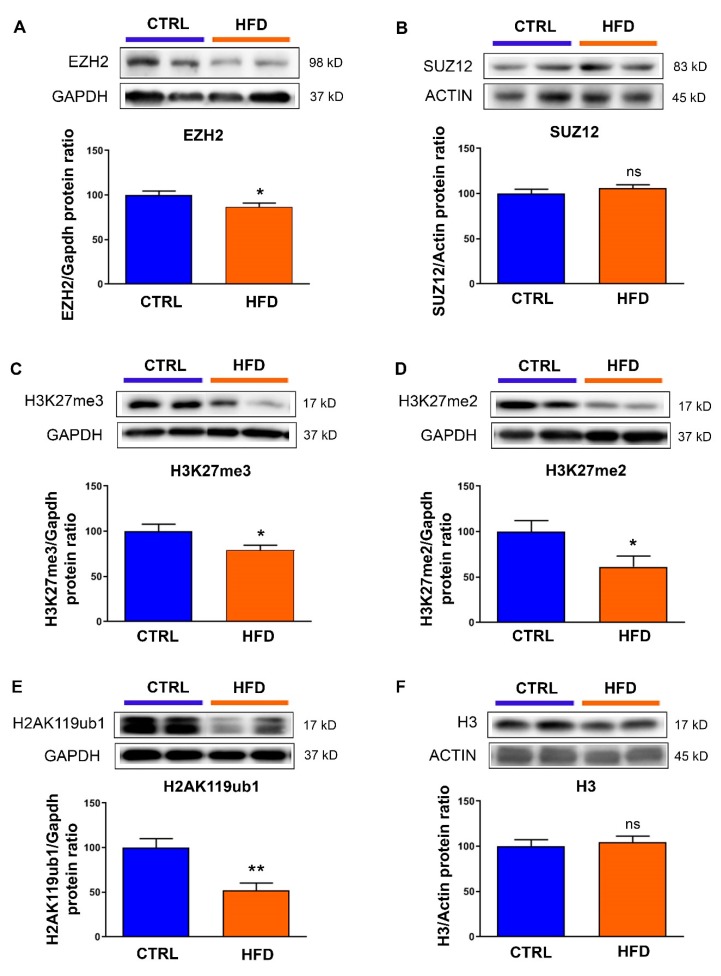
Effects of maternal exposure to high-fat diet on polycomb repressive complex 2. Protein levels of (**A**) enhancer zeste of homolog 2 (EZH2), (**B**) suppressor of zeste 12 (SUZ12), (**C**) histone H3 trimethyl lysine 27 (H3K27me3), (**D**) histone H3 dimethyl lysine 27 (H3K27me2), (**E**) histone H2A monoubiquitin lysine 119 (H2AK119ub1) and (**F**) histone H3 were analyzed in the heart from male rat at postnatal day 77 under control chow diet (CTRL) or high-fat diet (HFD). Representative immunoblot image is presented for each protein. Data are expressed as the mean ± SEM; *n* = 13 per group. * *p* <  0.05, ** *p*  <  0.01 compared to control, ns: not statistically significant.

We previously showed that maternal exposure to high-fat diet induces cardiac fibrosis and hypertrophy in male rat, at PND77, without alteration in the body weight [12]. Since polycomb repressive complex 2 (PRC2) has been described as an effector of environmental influences on gene expression and disease [22,23] and because alterations in PRC2 have been reported to induce cardiac hypertrophy and fibrosis, we wondered what could be the involvement of PRC2 in the programming of cardiac pathogenesis in these animals. In such an aim, using the same set of animals as previously described [12], we analyzed the expression of core components of the complex, enhancer of zeste homolog 2 (EZH2) (Figure 1A) and suppressor of zeste 12 (SUZ12) (Figure 1B). As such, we detected a significant decrease in EZH2 protein levels when animals were exposed to maternal high-fat diet (Figure 1A), whereas SUZ12 (Figure 1B) was not modified. To verify the impact of EZH2 deficiency on its histone marks, we analyzed the histone H3 di- and tri-methylation and, effectively, we found decreased H3K27me3 (Figure 1C) and H3K27me2 (Figure 1D) levels in the heart of the animals exposed to high-fat diet compared to chow diet. H3K27me3 can be recognized by PRC1, facilitate its recruitment and the monoubiquitination of histone H2A (H2AK119Ub1). Consistent with H3K27me3 alterations, H2AK119Ub1 levels were strongly down-regulated by maternal exposure to high-fat diet (Figure 1E). No change was detected in total histone 3 (H3) levels between the two groups of diet (Figure 1E).

### 3.2. Exposure to Maternal High-Fat Diet Induces Long-Term Alterations in DNA Methylation

**Figure 2:**
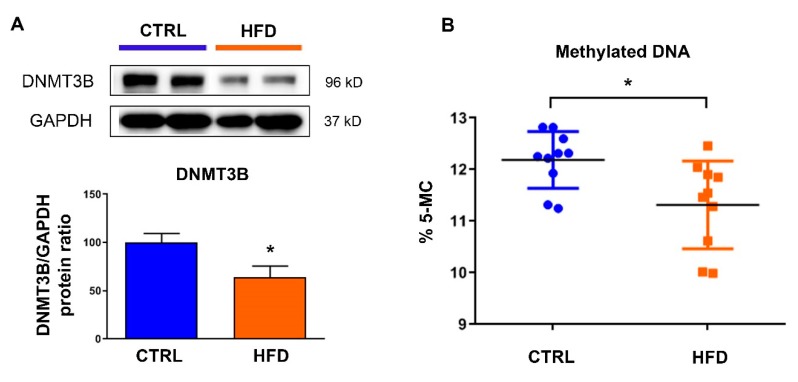
Effects of maternal exposure to high-fat diet on cardiac DNA methylation. (**A**) Protein levels of DNA methyl transferase 3B (DNMT3B) were analyzed in the heart from male rat at postnatal day 77 under control chow diet (CTRL) or high-fat diet (HFD). Representative immunoblot image is presented Data are expressed as the mean ± SEM; *n* = 13 per group. * *p*  <  0.05 compared to control. (**B**) Global DNA CpG methylation levels was measured in the heart of animals under control chow diet (CTRL) or high-fat diet (HFD). Data are expressed as the mean ± SEM; *n* = 10 per group. * *p*  <  0.05 compared to control.

H3K27me3 has been described as promoting DNA methylation and enhancing the chromatin repressive status. We thus evaluated the expression levels of the DNA methyltransferases (DNMTs) required for genome-wide de novo methylation, DNMT3B, in the heart and found decreased levels when animals were exposed to high-fat diet (Figure 2A). These alterations were associated with a global down-regulation in DNA methylation level (Figure 2B). 

### 3.3. Exposure to Maternal High-Fat Diet Derepresses Genes Involved in Fibrosis and Hypertrophy

**Figure 3:**
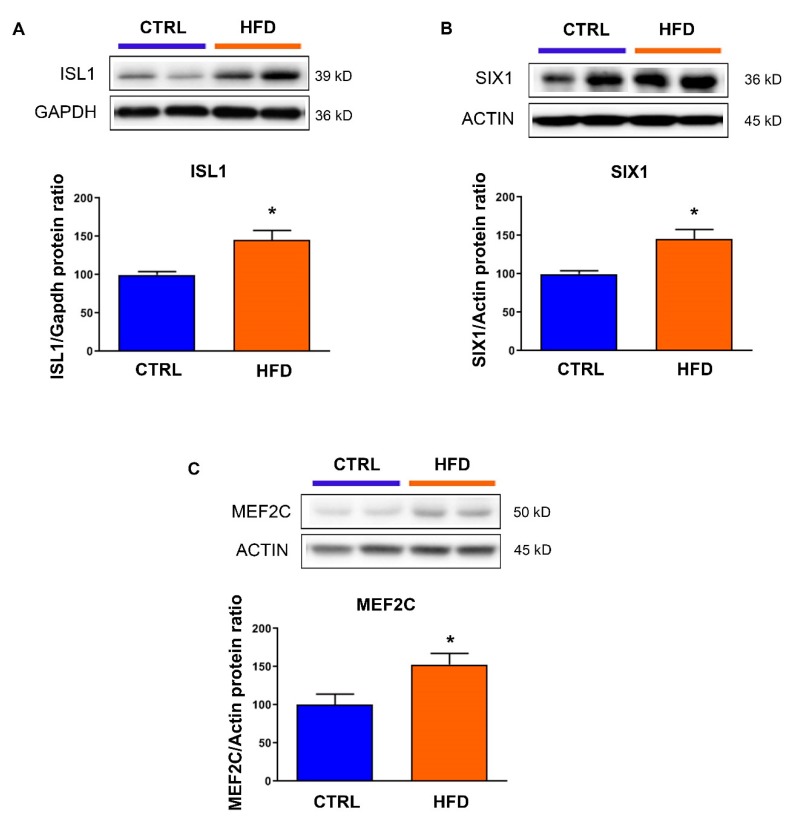
Protein levels of (**A**) isl lim homeobox 1 (ISL1), (**B**) six homeobox 1 (SIX1) and (**C**) mads box transcription enhancer factor 2, polypeptide C (MEF2C) were analyzed in the heart from male rat at postnatal day 77 under control chow diet (CTRL) or high-fat diet (HFD). Representative immunoblot image is presented for each protein. Data are expressed as the mean ± SEM; *n* = 13 per group. * *p*  <  0.05 compared to control.

To check whether the alterations in these repressive epigenetic marks were associated with altered expression of target genes involved in cardiac fibrosis and hypertrophy, we measured the expression level of isl lim homeobox 1 (ISL1) (Figure 3A), six homeobox 1 (SIX1) (Figure 3B) and mads box transcription enhancer factor 2, polypeptide C (MEF2C) (Figure 3C). Effectively, in animals exposed to high-fat diet, the protein levels of these genes regulated by H3K27me3 were increased compared to those exposed to chow diet.

## 4. Discussion

**Figure 4:**
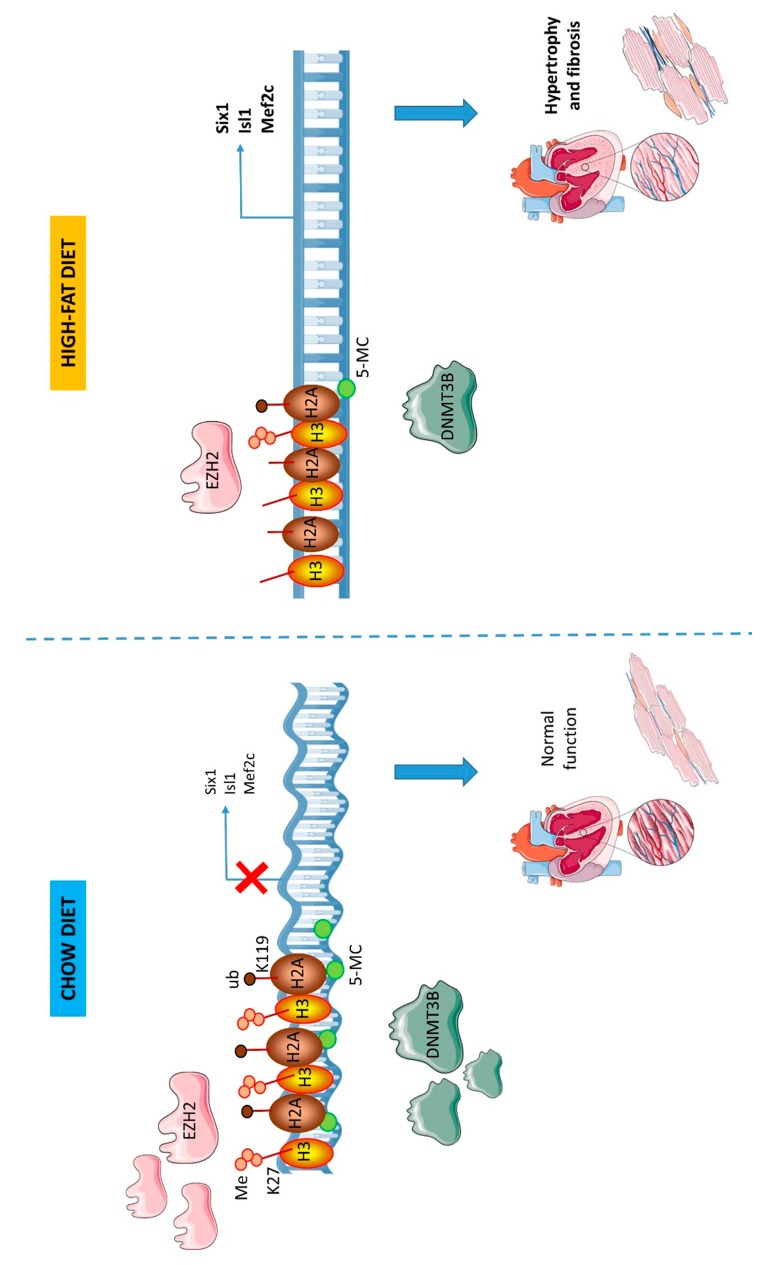
Effects of maternal exposure to high-fat diet on cardiac epigenetic repressive marks. Maternal exposure to high-fat diet induces long-term decrease in EZH2 levels. This leads to H3K27me2/3 down-regulation. At the same time, DNMT3B levels are decreased leading to down-regulated CpG methylation levels. These alterations mediate chromatin decompaction and subsequent activation of pro-hypertrophic and pro-fibrotic genes (*Six1, Isl1* and *Mef2c*).

We previously showed that maternal exposure to high-fat diet induces histological and molecular cardiac alterations in adult rats with hypertrophic and fibrotic patterns [12]. We highlighted the down-regulation of a microRNAs subset as a potential critical event in cardiac pathogenesis mediated by transforming growth factor beta (TGFβ) pathway dysregulation. In the origins of these alterations, we highlighted the important role of DiGeorge syndrome critical region 8 (*Dgcr8)*. However, the cardiac pathogenesis induced by maternal high-fat diet seems to involve additional mechanisms. Because a strong interplay exists between epigenetic regulations and because accumulation of alterations in different processes is critical in pathogenesis, we further investigated in the present study, alterations in other epigenetic mechanisms, aside non-coding RNAs. Collectively, our present data suggest that tissular cardiac alterations observed in the heart of animals exposed to maternal high-fat diet involve impaired repressive epigenetic mechanisms as well, including histone methylation and DNA methylation (summarized in Figure 4). 

Histones post-translational modifications and DNA methylation represent a bridge between environment and health. Furthermore, chromatin is described as a metabolic sensor linking regulation of gene expression to availability of nutrients and metabolites [24]. As such, maternal nutrition during early life leaves a “nutritional imprint” with long-term effects on the promotion of diseases [25,26]. In the etiology of chronic diseases, including inter- and transgenerational transmission [23], histone modifications and DNA methylation have been shown to play an important role through the setting of specific chromatin marks and their transmission throughout cell division during life. As such, in a model of early exposure to an environmental challenge (endocrine disruptors), we previously showed that diseases in adulthood were related to sustained impaired repressive histone mark, histone H3 trimethyl lysine 27 (H3K27me3), associated with deficiency in enhancer of zeste homolog 2 (EZH2) [22]. In a model of early exposure to dietary challenge, EZH2-mediated H3K27me3 has been highlighted as one epigenetic mechanism underlying nutritional programming of longevity [27]. Our data underlined the alterations in EZH2 as a potential epigenetic hallmark in the early origins of adult diseases related to early life challenges. The mechanisms through which EZH2 exert its effects on organ pathogenesis may involve multiple processes [22,28]. While most studies of EZH2 function have been performed in actively cycling cells such as tumoral cells or stem cells, less have been done in adult mammalian cells which cycle slowly, or adult cardiomyocytes which are mostly post-mitotic. In the heart, H3K27me3 and EZH2 have been shown to be essential for cardiomyocyte lineage-specification during embryogenesis [21,29]. The cardiac-specific inactivation of EZH2 during early cardiac development results in disruption of H3K27me3 deposition and lethal heart malformations. Those points suggest that the decreased EZH2 and subsequent alterations in histone marks induced by maternal exposure to high-fat diet, have profound consequences on heart development. In addition, null *Ezh2* mutant in the heart exhibits increased cardiomyocyte growth, with the derepression of non-cardiomyocyte gene programs, overexpression of pro-hypertrophic genes such as atrial natriuretic peptide (*Anp)* and beta heavy chain of myosin (*β-Mhc)* and pro-fibrotic genes such as sumo1-specific protease 1 *(Spp1)*, periostin (*Postn),* isl lim homeobox 1 (*Isl1*), six homeobox 1 (*Six1*) and mads box transcription enhancer factor 2, polypeptide C (*Mef2c*) [29]. Consistent with those findings, concurrent with EZH2 and H3K27me2/3 decrease, we observed increased expression of *Isl1*, *Mef2c* and *Six1* [21,29]. Interestingly, *Ezh2* knock out in the heart leads to the up-regulation of *Tgfβ3* [29], which might constitute an additional mechanism reinforcing the cardiac alterations in TGFβ signaling mediated by impaired DGCR8 in animals exposed to maternal high-fat diet [12]. In turn, TGFβ signaling has been shown to regulate epigenome through the interaction of receptor-regulated SMADs (R-SMADs) with epigenetic regulators including histones modifiers and to support, by this way, complex regulatory loops [30]. 

Not only, EZH2 represses gene through histone modification, but also methylates non-histone protein such as Gata-binding protein 4 (GATA4) [31]. GATA4 is a key transcription factor of heart development [32] and regulator of fibrosis [33]. GATA4 methylation by polycomb repressive complex (PRC) 2 results in reduced GATA4 transcriptional potency [31], indicating a potential additional role of EZH2 decrease in the cardiac fibrosis induced by maternal high-fat diet. Interestingly, global H3K27me3 levels have been found to decrease with ageing [34] and senescence [35]. Thus, the impact of EZH2 deficiency on cardiac ageing in the animals exposed to high-fat diet appears as an important point to address in further studies. Epigenetic mechanisms are tightly interrelated. Per se, H3K27me3 has been shown to recruit PRC1 complex and DNMTs to further induce histone H2A monoubiquitination (H2AK119ub1) and DNA methylation, particularly in the promoter regions. Those marks amplify gene repression through increased chromatin compaction or inhibition of RNA polymerase II. In our study, impaired H3K27me3 was associated with alterations in these marks. DNA methylation has been extensively studied and its central role has been highlighted notably in the control of tissue-specific gene expression, the regulation of differentiation and genome stability through the silencing of transposable element [36,37]. In the heart, DNA methylation regulates cardiomyocyte maturation and contractile function [38]. Alteration in DNA methylation has been shown to contribute to the metabolic remodeling in heart failure [39]. Interestingly, inhibitor of growth family member 1 (ING1), a transcriptional repressor which regulates *Dgcr8* expression [24], has been described as regulated by DNA methylation [25]. Even though our data indicating a global decrease in DNA methylation give a rationale for altered *Dgcr8* expression and down-regulated microRNAs levels that we previously observed in the animals exposed to maternal high-fat diet [12], further studies with a targeted analysis of *Dgcr8* and *Ing1* promoters should clarify this point. Aside methylation and ubiquitination, histone acetylation is one of the best characterized modifications and relies on the activity of histone acetyltransferases (HATs) [40] and histone deacetylases (HDACs). In the context of maternal exposure to high-fat diet, increased signaling involving the class IIa HDAC has been reported at postnatal day 10 in the heart [41]. The involvement of HATs and HDACs in cardiac hypertrophy and fibrosis is explained by their ability to regulate histone acetylation at the promoter region of *Gata4* and *Mef2c* and to associate with these transcription factors [42]. By this way, HATs and HDACs have been shown to regulate the expression of genes implicated in the control of stress-induced growth of the adult heart such as *Anp* and brain natriuretic peptide (*Bnp)* [41,43] as well as genes involved in fatty acid metabolism [41]. Importantly, the nutrition-dependent changes in DNA methylation and in histone marks can be gene specific. For instance, one study suggested that early exposure to nutritional excess affects histone acetylation in the newborn rat heart, in a way distinct from its influence on the gene-suppressive H3K27me3 mark [44]. It is thus of much interest to further determine the cardiac methylation profile and the “histone code” that could be associated with early exposure to nutritional challenge. These investigations might determine the epigenetic signature of cardiac dysfunctions induced by early exposure to nutritional excess. Also, in this study, global analyses were performed on the whole heart. Thus, it would be of interest to delineate more specifically the epigenetic changes in the different cardiac isolated cell populations. 

One main particularity of epigenetic marks is that they are reversible. For instance, the H3K27me3 mark is affixed by PRC2 and can be removed by histone demethylases such as lysine-specific demethylase 6A (KDM6A/UTX) and KDM6B/JMJD3. In addition, DNA demethylation can take place in a passive mode passively through the loss of DNA methylation activity, or actively through “reverse” enzymatic reaction driven by DNA methyltransferases in the absence of S-adenosyl-methionine, methyl CpG binding (MBD) proteins and the Ten-Eleven-Translocation (TET) family of enzymes [45,46]. Certain interventions such as caloric restriction [47], chemical drugs [48], and dietary supplementation [49] can reverse the inappropriate changes in chromatin. In the case of nutrients, levels of S-adenosylmethione and S-adenosylhomocysteine, which are substrates involved in DNA methylation, can be regulated through folate, vitamins B6 and B12, riboflavin, methionine, choline and betaine. For instance, dietary supplementation of the methyl donor folate increased both genomic and *p16* promoter DNA methylation in the aged mouse colon but not in the young, indicating that DNA methylation can be modified by diet in an age-dependent manner [50,51]. Bioactive food components such as retinoic acid [52], resveratrol [53], curcumin [54], sulforaphane [55] and polyphenols [56] have been shown to modify the activity of enzymes involved in histone modification. Thus, the targeting of EZH2 and/or DNMT3B through pharmacological or dietary approaches may represent powerful tools that could be used in therapy or in the prevention of heart dysfunctions induced by early exposure to nutritional excess. 

## 5. Conclusions

Altogether, our study indicates that early developmental life represents a sensitive period, when exposure to nutritional excess shapes the lifelong cardiac epigenetic marks through, at least in part, impaired polycomb repressive complex and DNA methylation, mediating cardiac dysfunctions.

## Supplementary Materials

The following are available online at https://www.mdpi.com/2072-6643/12/1/181/s1, supplementary information.
