# Supplementary material for: Maternal Exposure to High-Fat Diet Induces Long-Term Derepressive Chromatin Marks in the Heart
Source: Nutrients. 2020 Jan 9;12(1):181. doi: 10.3390/nu12010181 (PMC7019950; doi:10.3390/nu12010181)
Supplement: Supplementary file 1 [file nutrients-12-00181-s001.pdf]

SUPPLEMENTARY INFORMATION

	CTRL diet (Safe R03)	High-fat diet (Ssniff EF)
**Gross energy (per kg)**	14.21 MJ	24.4 MJ

Metabolizable energy:

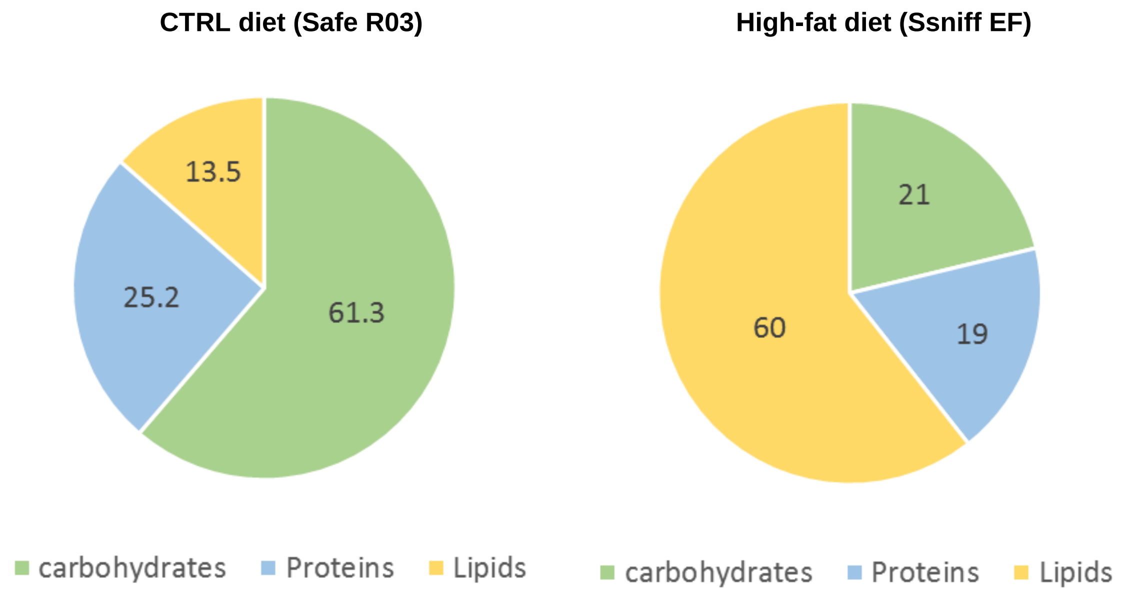

FATTY ACIDS

Fatty acid	CTRL diet (Safe R03) (per kg)	High-fat diet (Ssniff EF) (per kg)
C16:0	7600 mg	80600 mg
C16:1	500 mg	7800 mg
C17:0	nd	3800 mg
C18:0	1500 mg	56100 mg
C18:1	10000 mg	121300 mg
C18:2	25000 mg	23700 mg
C18:3	2800 mg	3300 mg

AMINO ACIDS

Amino acids	CTRL diet (Safe R03) (per kg)	High-fat diet (Ssniff EF) (per kg)
Lysine	11500 mg	19600 g
Methionine	4400 mg	8300 mg
Cystine	3200 mg	4600 mg
Thryptophan	2600 mg	3100 mg
Arginine	14000 mg	8800 mg
Glycine	12000 mg	5000 mg
Threonine	nd	10700 mg
Glutamic acid	nd	54100 mg
Aspartic acid	nd	17900 mg
Proline	nd	27600 mg
Alanine	nd	7900 mg
Serine	nd	14300 mg
Histidine	nd	7600 mg
Valine	nd	16400 mg
Isoleucine	nd	12500 mg
Leucine	nd	23600 mg
Phenylalanine	nd	12900 mg

nd: not determined

VITAMINS

Vitamins	CTRL diet (Safe R03) (per kg)	High-fat diet (Ssniff EF) (per kg)
Vitamin A	14000 IU	15000 IU
Vitamine D3	2000 IU	1500 IU
Vitamine E	100.5 mg	150mg
Vitamine B1	8 mg	16 mg
Vitamine B2	13 mg	16 mg
Vitamine B5	15 mg	18mg
Vitamine B12	0.02 mg	0.03 mg
Vitamine K3	5.7 mg	nd
Vitamine K	nd	20 mg
Folic acid	0.5 mg	19 mg
Biotin	0.1 mg	0.310 mg
Choline	2100 mg	2300 mg
Nicotinic acid	90 mg	45 mg
Panthotenic acid	nd	55 mg
Inositol	nd	80 mg

MINERALS AND TRACE ELEMENTS

Composition (per kg)	CTRL diet (Safe R03)	High-fat diet (Ssniff EF)
Phosphorous	5900 mg	6500 mg
Calcium	8200 mg	10500 mg
Sodium	2800 mg	2000 mg
Magnesium	2000 mg	1700 mg
Potassium	8600 mg	10000 mg
Iron	280 mg	139 mg
Manganese	90 mg	82 mg
Zinc	64 mg	56 mg
Copper	18 mg	12 mg
Iodine	nd	0.97 mg
Selenium	nd	0.13 mg
Cobalt	nd	0.13 mg
Cl	4100 mg	nd

nd: not determined
